# Effects of an animated educational video on knowledge of cell-free DNA screening among Thai pregnant women: a randomized control trial

**DOI:** 10.1186/s12884-023-06170-8

**Published:** 2023-12-11

**Authors:** Nutta Nintao, Jittima Manonai, Rujira Wattanayingcharoenchai, Sommart Bumrungphuet, Wirada Hansahiranwadee, Wirada Dulyaphat, Werapath Somchit, Duangrurdee Wattanasirichaigoon, Maneerat Prakobpanich, Chayada Tangshewinsirikul

**Affiliations:** 1 Department of Obstetrics & Gynaecology, Faculty of Medicine Ramathibodi Hospital, Mahidol University, Bangkok, Thailand; 2 Division of Maternal-Fetal Medicine, Department of Obstetrics & Gynaecology, Faculty of Medicine Ramathibodi Hospital, Mahidol University, Bangkok, Thailand; 3 Division of Medical Genetics, Department of Pediatrics, Faculty of Medicine Ramathibodi Hospital, Mahidol University, Bangkok, Thailand

**Keywords:** Animated educational video, Cell-free DNA, NIPT, Prenatal counseling

## Abstract

**Background:**

In developing countries, pregnant women have insufficient knowledge about cell-free DNA screening. Reports from developed countries have found that various tools in prenatal genetic counseling can improve the knowledge of pregnant women who undergo cell-free DNA screening. Data are limited from developing countries where women have different baseline socio-educational backgrounds. The objective of this study was to compare the effects of an animated educational video combined with traditional counseling versus traditional counseling alone in changing pregnant women’s knowledge of cell-free DNA screening.

**Methods:**

This study was a randomized control trial at an antenatal clinic. Eligible subjects who were Thai pregnant women, were randomized to either view or not view the 4-minute animated educational video explaining cell-free DNA screening. Both groups received traditional counseling. The women were asked to complete a Thai questionnaire assessing knowledge of the screening before and after intervention. The questionnaire consisted of three sections: demographic data of the research participants and their existing awareness about cell-free DNA testing; performance and limitations of cell-free DNA screening; and participants’ attitudes toward the positive screening. Primary outcome was the change in knowledge scores. Secondary outcomes were attitudes toward positive screening test, levels of satisfaction with counseling, and screening acceptance rates.

**Results:**

Data from 83 women in the video group and 82 in the non-video group were analyzed. The knowledge score (range 0–18) change after counseling was significantly higher in the video group than the non-video group (+ 7.1 ± 3.3 vs + 4.2 ± 2.5; *p* = 0.03). There were no significant differences in attitudes toward positive screening test (*p* = 0.83), levels of satisfaction (*p* = 0.24), or screening acceptance rates (*p* = 0.15) between the groups.

**Conclusions:**

Adding the video to traditional counseling was better than traditional counseling alone in improving pregnant women’s knowledge about cell-free DNA screening.

**Trial registration:**

The study was retrospectively registered with the Thai Clinical Trials Registry (TCTR20210917001, 17/09/2021).

## Background

Noninvasive prenatal screening based on cell-free DNA sequencing was first established in 1997 [1]. Its use started to be implemented in prenatal settings in 2011, and the technique is now used worldwide [2–5]. It is recognized as the most powerful tool for screening for common fetal aneuploidies because of its high sensitivity and specificity [2–5]. However, the test has some limitations and can result in both false positive and false negative results [2–5]. Unless pregnant women receive adequate information, the test results may cause anxiety and, potentially, adverse pregnancy outcomes [6–9]. Therefore, professional guidelines recommend offering cell-free DNA screening to all pregnant women in the context of informed decision-making [6–9]. Providing adequate information during pretest counseling improves knowledge and informed choices among pregnant women [2, 10, 11].

Thailand is a developing country with a national policy for prenatal screening and diagnosis [12–16]. Cell-free DNA screening was introduced to the country in 2012. Since then, it has generally been offered by health-care professionals and accepted by pregnant women as a primary screening test option in routine prenatal care, even though it is not covered by Thailand universal health coverage [17, 18]. However, previous studies have found that Thai pregnant women have insufficient knowledge of this type of screening and that this knowledge differs from that of people in developed countries [19–22].

Reports from developed countries have found that various tools in prenatal genetic counseling, such as educational apps and videos as well as low literacy decision aids, can improve the knowledge of pregnant women who undergo cell-free DNA screening [23–26]. However, data are limited from Thailand and other developing countries where women have different baseline socio-educational backgrounds. The animated video are videos created with original designs, drawings, illustrations, and computer-generated effect that have been produced in an eye-catching way, including sound to narrate a message. Previous study demonstrated that animated educational videos may convey complex information in a straightforward manner and increased patient knowledge [27, 28].

Therefore, the primary aim of this study was to compare the effectiveness of an animated educational video combined with traditional counseling versus traditional counseling alone in changing pregnant women’s knowledge of cell-free DNA screening. The secondary aims were to assess attitudes toward positive screening test, levels of satisfaction with counseling, and rates of acceptance of cell-free DNA screening after counseling.

## Methods

### Study design

This randomized controlled trial was conducted between September 2021 and March 2022 at the antenatal care clinic, Faculty of Medicine Ramathibodi Hospital, Mahidol University, Bangkok, Thailand. The protocol was approved by the Ramathibodi Hospital Institutional Review Board (COA, MURA2021/748) and complied with the Declaration of Helsinki. The study was registered with the Thai Clinical Trials Registry, TCTR20210917001 (17/09/2021).

### Participants

All singleton pregnant women aged 18 years and over and capable of reading and understanding Thai, with a gestational age of 14 weeks or less, who first visited our antenatal care clinic were routinely offered to have cell-free DNA testing without asking about their prior intention. They were consecutively invited to enroll in the study by an author (NN). All the participants provided their written informed consent before randomization.

### Randomization

A random allocation sequence was carried out in blocks of four by a statistician and the results placed in sealed sequentially numbered envelopes and labeled. A perinatal research nurse (MP) generated randomization allocation sequence and assigned participants to view (video group) or not view (non-video group) an animated educational video. An author who enrolled participants (NN) and the counselors, who included standardized and trained obstetrics and gynecology residents and maternal–fetal medicine fellows and staff, were blinded to the randomization sequence.

### Baseline characteristics

Before the intervention, all the participants completed a Thai language questionnaire to access their prior knowledge about cell-free DNA screening and their attitudes toward positive results. The questionnaire had previously been developed and validated by Chalopagorn and Manotaya, who gave permission for its use in this study [22]. The Cronbach’s alpha for the questionnaire was 0.702. The questionnaire consisted of five pages, which were separated into three sections. The first section focused on the participants’ demographic data, including maternal age, religion, education, occupation, gravida, and history of previous fetal anomalies, and queried whether the participants had heard about cell-free DNA testing. The second section asked about the performance and limitations of cell-free DNA screening. It contained 15 items of True, False, and I don’t know question, and three multiple-choice questions with one best response. The last section aimed to determine the participants’ attitudes toward positive screening test from cell-free DNA testing using a five-point Likert-type scale, where “1″ indicated strongly unconcerned and “5″ denoted strongly concerned.

### Interventions

Following the completion of the questionnaire, each participant received a session of “traditional counseling”, which included individual counseling and an information sheet for them to read. The individual counseling was face-to-face structured pretest counseling from one of the counseling physicians about the characteristics of common autosomal trisomies, testing options including maternal serum screening, cell-free DNA screening and prenatal diagnosis; and details of cell-free DNA screening. Given genetic counsellor profession did not exist in Thailand, all prenatal genetic counseling in antenatal clinic has been delivered by standardized and trained obstetrics and gynecology residents and maternal–fetal medicine fellows under supervision of the staff, and in some occasion by the staff themselves. Each counseling session took place in an examination room and lasted approximately 10 minutes. All the participants were subsequently given a Thai language information sheet on cell-free DNA screening. The information sheet was developed by maternal–fetal medicine staff (CT, SB, WH, WD, WS), which covered characteristics of common autosomal trisomies, the fetal conditions that can be detected via cell-free DNA screening, the performance and limitations of such screening, the clinical significance of the different screening results, and further management following positive results. The participants were given 10 minutes to read the information sheet in a designated area.

**Fig. 1:**
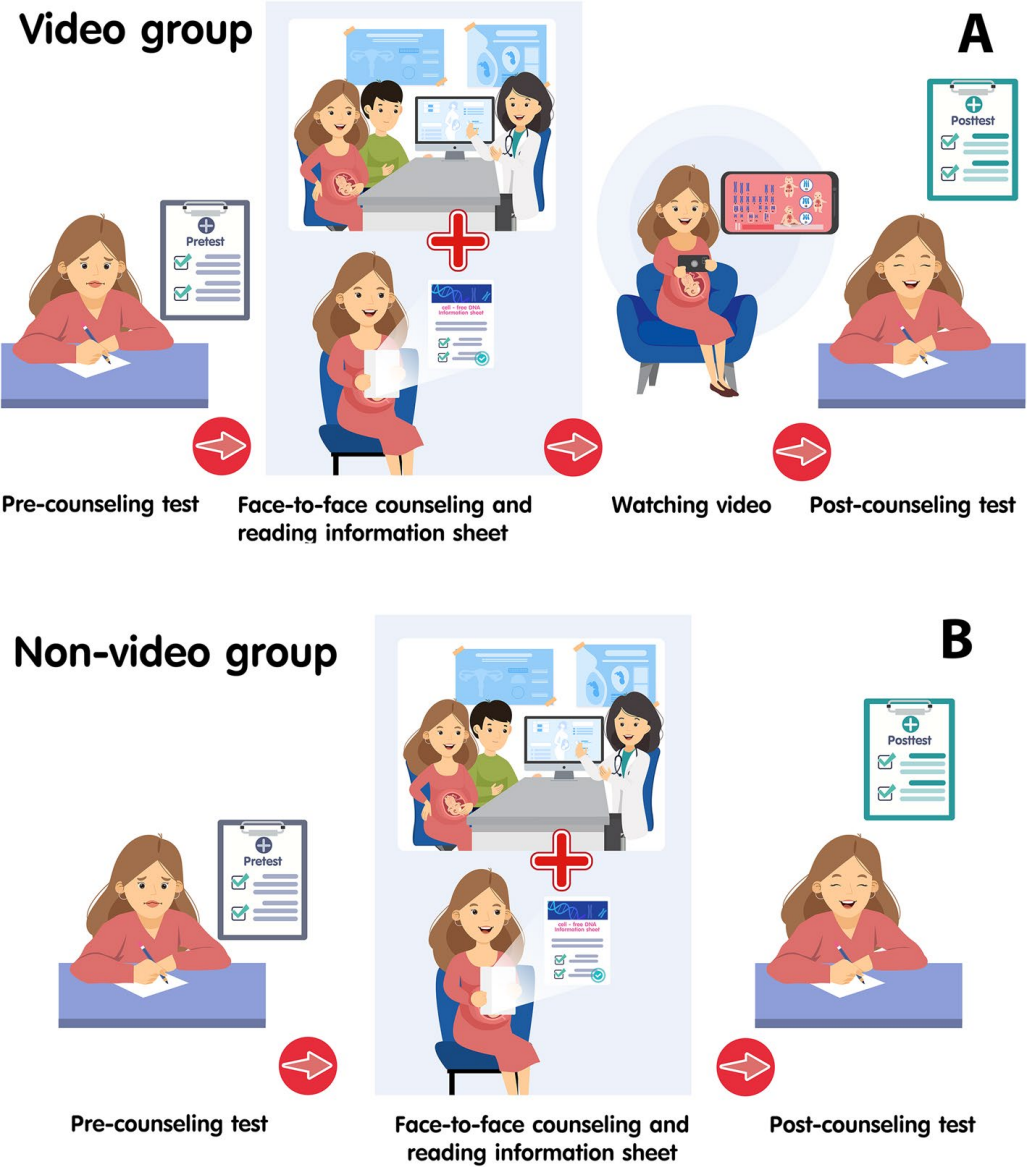
Pretest counseling methods in video group (**A**) and non-video group (**B**)

After the traditional counseling, the participants in the video group were given a video’s link (https://youtu.be/HCsvVtKV3ck?si=Md2L6yvV-mAz0Dx7) to watch a 4-minute animated educational video, using their mobile phone in the designated area. The video was produced in the Thai language and was developed, scrutinized, and standardized by the principal investigator (NN), maternal–fetal medicine staff (CT, SB, WH, WD, WS), and a pediatric geneticist (DW). The 4-minute animated educational video provided basic information on common autosomal trisomies and essential details of the cell-free DNA screening. The information in the video was similar to that in the information sheet. Pretest counseling methods in video group and non-video group is shown in Fig. 1.

### Outcomes measures

Primary outcome in this study was the change in knowledge scores. Secondary outcomes were attitudes toward positive screening test, levels of satisfaction with counseling, and screening acceptance rates.

After the pretest counseling and on the same day as the counseling, all the participants completed the same questionnaires and gauged their levels of satisfaction by using five-point Likert-type scale.

In the second section of questionnaires, true, false, I don’t know questionnaire, if the participants chose correct answer, they would be given a score of “1”, in contrast if the participants chose incorrect answer or “I don’t know,” they would be given a score of “0” for that question. In the three multiple-choice questions with one best response, the score of “1” and “0” was given for a correct and incorrect answer, respectively. In sum, the maximum score was 18/18.

Results from the attitude towards positive results and satisfaction were dichotomized by combining level of concerning or level of satisfaction of “strongly concerned/very satisfied” and “concerned/satisfied” into one category labeled “Concerned/Satisfied”; and “neutral/average”, “unconcerned/slightly satisfied” and “strongly unconcerned/not satisfied” into another category labeled “Unconcerned/Not satisfied”.

The women were then told that they could request additional explanations from the counselors to ensure that they clearly understood the information about cell-free DNA screening and were able to make their own decisions about whether to accept this screening option. Finally, the educational levels of the counselors were documented, and the time between the pre- and post-counseling test was measured.

### Sample size estimation

We conducted a pilot study to assist with the sample size estimation. Twenty pregnant women who volunteered to participate were randomized to complete the questionnaire before the pretest counseling. Their mean knowledge score was 7.1 ± 4.6 (Full scores: 18). After traditional counseling, the mean score increased to 9.7 ± 4.6, which was about 36% increase. We predicted that watching the video may lead to double incremental knowledge score (70% increase) at 12.1 ± 4.6. Assuming a two-sided alpha of 0.05 and 90% power to detect a significant difference, this meant we needed 80 participants per group. We estimated the potential for 10% data loss and therefore aimed to enroll 88 participants in each group to give a total of 176 participants.

### Statistical analysis

Categorical variables were defined as the number (percent) and compared using chi-square or Fisher’s exact test. Parametric continuous variables are expressed as the mean ± standard deviation and were compared using Student’s t-test. A *P*-value *<* 0.05 was considered statistically significant. Statistical analyses were performed using STATA, version 17 (STATA Corp, College Station, TX, USA).

## Results

**Fig. 2:**
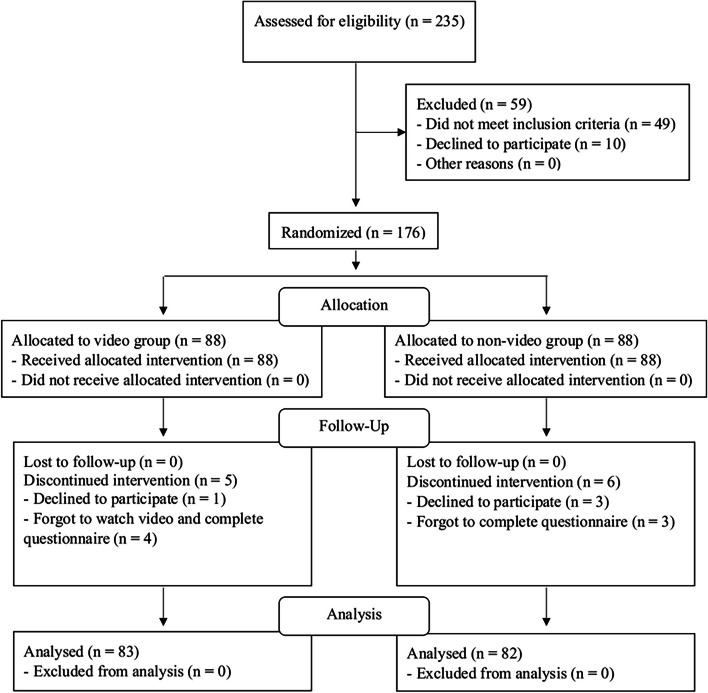
CONSORT 2010 flow diagram for ‘Effects of an animated educational video on knowledge of cell-free DNA screening among pregnant women: a randomized control trial’

Among 235 pregnant women who were approached, 49 did not meet the inclusion criteria and 10 declined to participate, leaving 176 to be enrolled in the study. They were randomized into either the video or non-video groups. According to the principles of “intention to treat analysis”, participants are not to be excluded when they do not complete the intervention. However, in case of missing data regarding the outcome, they would be excluded. (five participants from the video group and six from the non-video group). The CONSORT diagram of the study is shown in Fig. 2.

Both groups had comparable demographic data. The educational levels of the counselors and the time between the pre- and post-counseling test were not significantly different between the two groups (Table 1).

**Table 1:** Demographic data of participants in the video and non-video groups

Characteristic	Video group *n* = 83 (%)	Non-video group *n* = 82 (%)	*p*-value
Age (years), (mean ± SD)	30.5 ± 10.6	31.8 ± 9.2	0.53
Gestational age (day), (mean ± SD)	83.4 ± 1.3	82.2 ± 2.5	0.23
Religion			0.98
Buddhist	78 (94.0)	77 (93.9)	
Muslim	5 (6.0)	5 (6.1)	
Level of education			0.92
Below bachelor’s degree	26 (31.3)	26 (31.7)	
Bachelor’s degree	48 (57.8)	49 (59.8)	
Above bachelor’s degree	9 (10.9)	7 (8.5)	
Occupation			0.42
Government officer	37 (44.6)	30 (36.6)	
Company employee	23 (27.7)	24 (29.3)	
Other employee	4 (4.8)	3 (3.7)	
Self-employed	9 (11.1)	13 (10.9)	
Unemployed	8 (9.6)	5 (6.1)	
Other	2 (2.4)	7 (8.5)	
Habitation			0.69
Bangkok	62 (74.7)	59 (72.0)	
Other	21 (25.3)	23 (28.0)	
Medical complications			0.45
No	65 (78.3)	68 (82.9)	
Yes	18 (21.7)	14 (17.1)	
Gravida			0.07
Gravida 1	40 (48.2)	27 (32.9)	
Gravida 2	27 (32.5)	25 (30.5)	
Gravida 3	14 (16.9)	24 (29.3)	
Gravida ≥4	2 (2.4)	6 (7.3)	
Previous child with anomaly/genetic disorder			0.68
No	81 (97.6)	79 (96.3)	
Yes	2 (2.4)	3 (3.7)	
Cf-DNA counseling in a previous pregnancy			0.98
No	77 (92.8)	76 (92.7)	
Yes	6 (7.2)	6 (7.3)	
Have heard about cf-DNA screening			0.82
No	65 (78.3)	63 (76.8)	
Yes	18 (21.7)	19 (23.2)	
Counselor level			1.00
Resident	55 (66.3)	54 (65.9)	
Staff members and fellow MFM	28 (33.7)	28 (34.2)	
Time between pre- and post-counseling test (mean ± SD) (minutes)	72.4 ± 74.4	84.7 ± 74.3	0.99

cf., cell-free; MFM, maternal–fetal medicine; OBGYN, obstetrics and gynecology

The mean ± SD knowledge scores in the two groups before counseling were comparable: 7.4 ± 3.1 in the video group vs 7.3 ± 3.2 for the non-video group (*p* = 0.74). After counseling, the mean ± SD knowledge scores were significantly higher than the baseline knowledge scores in both groups (video group 14.5 ± 2.7, *p* < 0.01 vs non-video group 11.5 ± 3.3, *p* < 0.01). However, the video group showed a significant improvement in their mean knowledge score (+ 7.1 ± 3.3) compared to the non-video group (+ 4.2 ± 2.5; *p* = 0.03) (Table 2).

**Table 2:**
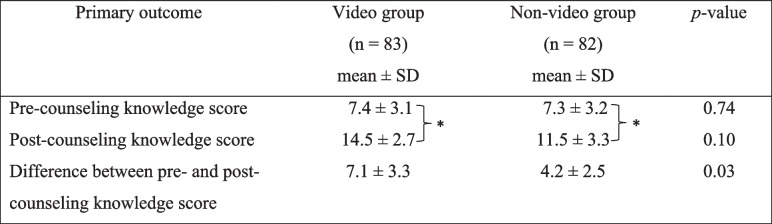
Comparison of knowledge scores between the video and non-video group.

* *p* < 0.01

Before counseling, a similar percentage of the participants in each group had correct knowledge about fetal conditions that could be detected via cell-free DNA screening. After counseling, the percentage of participants with the correct knowledge was significantly increased in both groups (*p* < 0.01). However, the video group gained a significantly higher percentage of participants who correctly identified the conditions that could not be detected by cell-free DNA screening (Table 3).

**Table 3:**
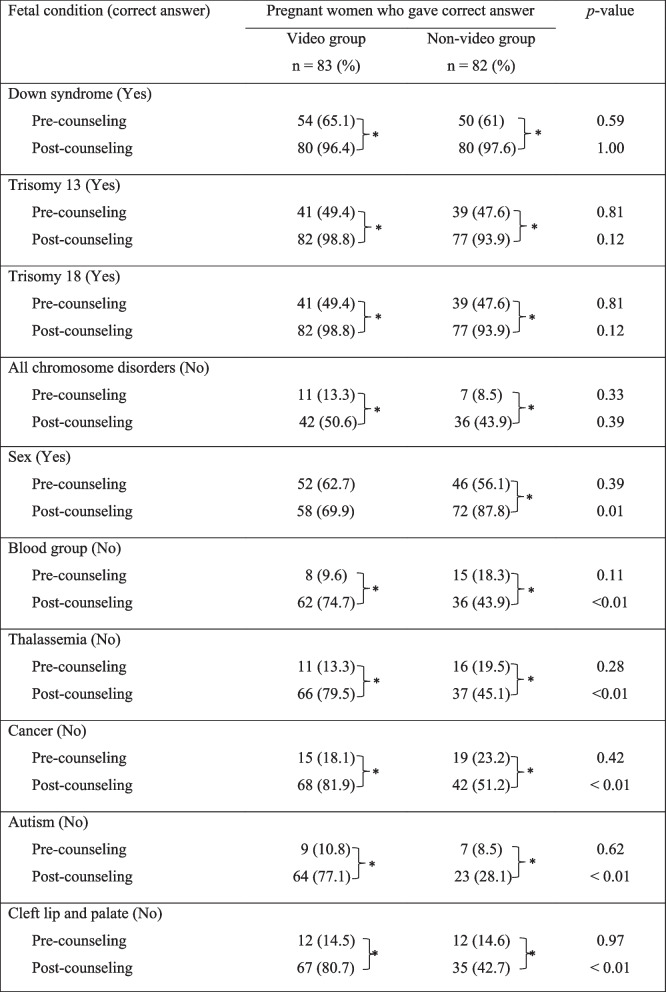
Comparison of pregnant women’s knowledge about fetal conditions detected by cell free- DNA screening, between groups.

* *p* < 0.01

The two groups, at baseline, showed comparable percentage of participants who knew about the performance and limitations of cell-free DNA screening. After counseling, there was a higher percentage of pregnant women in the video group who understood about false positive rate, limitation of using positive cell-free DNA screening for the decision of termination of pregnancy, possibility of false negative screening for Down syndrome, and avoidance of cell-free DNA screening in individuals with recent blood transfusion (Table 4).

**Table 4:**
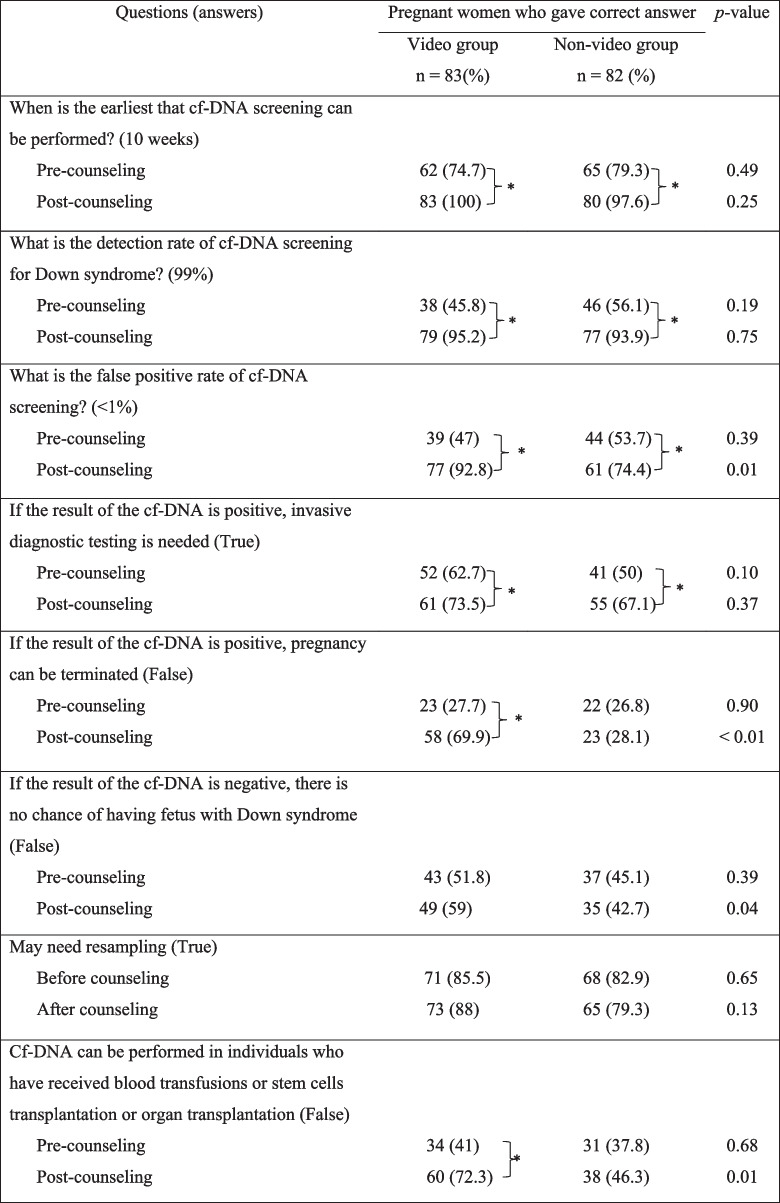
Comparison of pregnant women’s knowledge about performance and limitations of cell-free DNA screening, between groups.

Cf, cell-free

*p < 0.01

In both groups, there was no statistically significant difference in concern about positive cell-free DNA screening before counseling, as well as after the counseling (*p* = 0.83). The level of satisfaction with counseling was high, but this was not significantly different between the groups (*p* = 0.24) (Table 5). Lastly, the rate of acceptance of cell-free DNA screening was slightly over half and comparable between the two groups (66.3% vs 54.9%; *p* = 0.15).

**Table 5:** Comparison of attitude toward receiving a positive result and counseling satisfaction level, between groups

	Video group *n* = 83 (%)	Non-video group *n* = 82 (%)	*p*-value
Attitude toward receiving a positive results			
Before counseling			0.77
Concerned	76 (91.6)	74 (90.2)	
Unconcerned	7 (8.4)	8 (9.8)	
After counseling			0.83
Concerned	73 (88)	73 (89)	
Unconcerned	10 (12.1)	9 (11)	
Level of satisfaction with counseling method			0.24
Satisfied	75 (90.4)	78 (95.1)	
Not satisfied	8 (9.6)	4 (4.9)	

## Discussion

We found that pregnant women’s knowledge of cell-free DNA screening increased after pretest traditional counseling and even higher when an animated educational video was added. The attitude toward positive screening test, level of satisfaction to the counseling, and screening acceptance rate were not statistically different between the video and non-video group.

Data from the present study consistent with previous studies conducted in developed countries [23–26]. The result underlines the importance of pretest genetic counseling for all pregnant women as this facilitates understanding before decision-making.

Before counseling, the majority of the participants had misunderstanding that some non-chromosomal disorders, especially thalassemia, could be detected via cell-free DNA screening., which is in agreement with previous study [19, 22]. Thalassemia is the most prevalent monogenic disorder in Thai population with carrier rate of 40% (alpha and beta thalassemia) and incidence of 1 in 100 births, if no intervention is provided [29]. Prenatal carrier screening for thalassemia has been offered to all pregnant women as part of national policy and public health services across the country, with free-of-charge since 1992 [29]. To explore if the participants might have had misunderstanding between carrier screening for thalassemia and cell-free DNA trisomy screening, we included thalassemia in to our questionnaire.

Prior to the counseling, one-fourth of the participants had correct knowledge that termination of pregnancy should not be considered solely based on cell-free DNA positive results and the traditional counseling had little effect on increasing this knowledge to the participant (Table 4), while this knowledge was significantly improved after the video was added [20]. We suggest that, it is important to emphasize the chance of a false positive result and the need for confirmation test.

Although individual counseling and information sheet are generally considered sufficient for genetic counseling, we thought that a video could add some advantages as follow: 1) images-linked to written or spoken text in animated video can expand patients’ comprehension and simplify complex technical terms about cell-free DNA screening, as a result an increase of patients’ knowledge [30]; 2) the video can be watched multiple times as needed; 3) if the video works well, we then can use the video for mass education to pregnant women and women who are planning for their pregnancies; 4) the use of the video can also help to standardize communication process, increase effective communication, and reduce the gap from interpersonal variations among the counseling providers [31–34]. Based on our experience from the present study, the video counseling was added to our work instruction and used in our antenatal care clinic. The video is used to complement rather than replace face-to-face counseling.

The acceptance rate of cell-free DNA screening in this study was high when compared with previous studies [10, 19, 35]. Several factors may influence patients’ decisions to take cell-free DNA screening, including knowledge, cost, maternal age, educational level, test accuracy, and the likelihood of terminating a pregnancy [35–39]. Further studies are needed to understand patients’ attitudes and the factors influencing the rate of acceptance of cell-free DNA screening in this area.

To the best of our knowledge, this is the first study to compare the effectiveness of two methods of providing counseling about cell-free DNA screening in a developing country. It was a randomized control trial that all the counselors were blinded to the randomization sequence, which could decrease selection bias. The questionnaire was originally developed and validated using Thai language, therefore we believe that outcome measures were socio-culturally fitting. The video could be considered to use in other hospitals in Thailand or translated into other languages for use in other countries where the patient characteristics are similar.

This study also had some limitations. Over half the participants had at least a bachelor’s degree. Although most of the pregnant women in our antenatal clinic were unaware of cell-free DNA screening before counseling, their high educational level may have affected their acquisition of knowledge about this screening technique [40]. In addition, the study was conducted in a single center, and genetic counseling was provided by trained residents, fellows, and staff. The same results may therefore not be obtainable in other contexts.

## Conclusion

The animated educational video combined with traditional counseling was better than traditional counseling alone in increasing pregnant women’s knowledge of cell-free DNA screening and therefore it should be considered as part of the pretest counseling.

## Data Availability

The datasets generated and/or analyzed during the current study are available from the corresponding author based on a direct request.
